# Supplementary figures and images for: Salivary Antibody Responses to Two COVID-19 Vaccines following Different Vaccination Regimens
Source: Vaccines (Basel). 2023 Mar 28;11(4):744. doi: 10.3390/vaccines11040744 (PMC10146373; doi:10.3390/vaccines11040744)

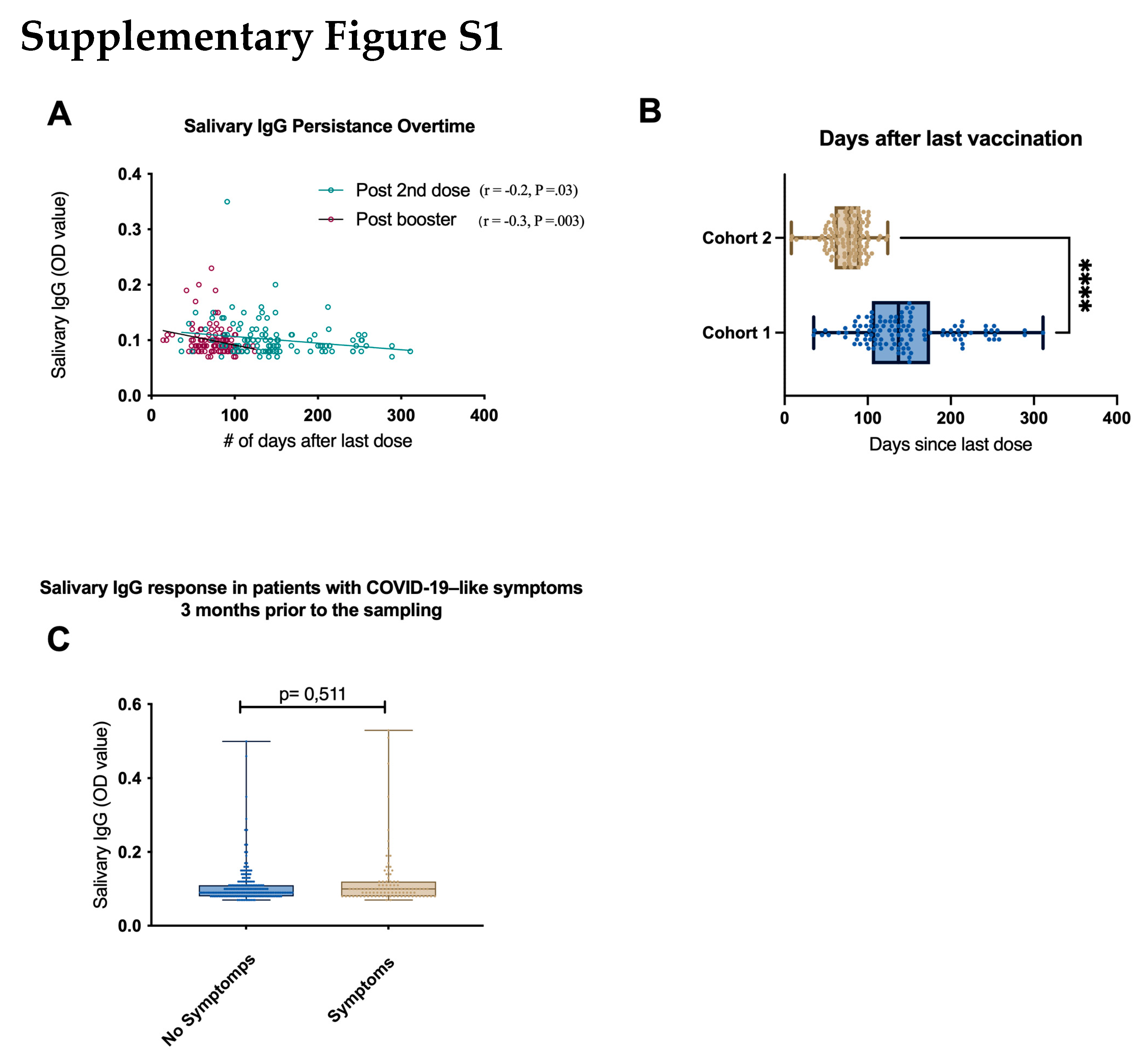

Supplement: Supplementary file 1 [file vaccines-11-00744-s001.zip › vaccines-2276392-supplementary.tiff]
